# Perceived organizational support and organizational citizenship behavior–A study of the moderating effect of volunteer participation motivation, and cross-level effect of transformational leadership and organizational climate

**DOI:** 10.3389/fpsyg.2023.1082130

**Published:** 2023-02-09

**Authors:** Jui-Chung Kao, Cheng-Chung Cho, Rui-Hsin Kao

**Affiliations:** ^1^Institute of Marine Affairs and Business Management, National Kaohsiung University of Science and Technology, Kaohsiung, Taiwan; ^2^Department of Ocean and Border Management, National Quemoy University, Jincheng, Taiwan

**Keywords:** perceived organizational support, volunteer participation motivation, organizational citizenship behavior, transformational leadership, organizational climate

## Abstract

The purpose of this study is to examine the relationship between perceived organizational support and organizational citizenship behavior, and to explore the moderating effect of volunteer participation motivation on the relationship between the variables, as well as the cross-level effect of transformational leadership and organizational climate. In this study, the front-liners of Taiwan’s National Immigration Agency were the study subjects. A total of 289 employee questionnaires were filled out and returned. It was found that employees’ POS had a positive effect on OCB, while volunteer participation motivation had a moderating effect on the relationships between the variables. Furthermore, transformational leadership and organizational climate were found to have a cross-level effect on enhancing employees’ POS, boosting their motivation to volunteer, and triggering more OCB in employees. The results of this study provide the organization with development measures to encourage its employees to convey more OCB, and improve their service performance. Moreover, based on research evidence that an organization encourages employees to actively participate in voluntary work, and should promote cooperation between the employees and the public by enhancing their sense of public responsibility, improving their services to the public, creating a harmonious work climate for the employees, and offering more opportunities for the public to engage with the employees.

## 1. Introduction

The old-fashioned bureaucratic model takes the perspective of administrative convenience in public service provision, whereas the customer-oriented service model emphasizes that organizations should focus on customers and provide services or products that meet the needs of customers (Koehler and Pankowski, 1996). The implication of social exchange theory (social exchange theory) shows that perceived organizational support (POS) can promote organizational citizenship behavior (OCB) of employees (Abdullah and Wider, 2022). Despite being difficult to normalize OCB in a formal organizational management system, OCB can be indirectly promoted or controlled by a non-formal approach. One can also use positive environmental conditions to reinforce employees’ OCB (Kao, 2017). Therefore, regarding Taiwan’s National Immigration Agency, the organization may be able to trigger employees’ OCB through POS or altruistic motivation.

Although studies have shown that POS could elicit more OCB in employees, the degree of employees’ POS is not positively associated with the OCB frequency. For example, Thompson et al.’s (2020) study found that even if women do not have higher perceived organizational support, they feel more obliged to engage in extra-role behaviors than men. In addition, the level of individual-organization fit (Jehanzeb, 2020) and the violations of psychological contract will also affect employees’ OCB performance (Shaheen et al., 2016; Wang et al., 2023). Consequently, a high level of POS does not necessarily trigger more OCB; the association between POS and OCB is affected by many factors, including personal behavior. To encourage employees to autonomously meet extra-role requirements and contributions to help the organization achieve its goal, it is important to determine if there are other reinforcements beyond the POS. According to the altruistic component of OCB, this study suggests that in addition to improving POS, organizations can increase employee OCB by increasing their motivation to volunteer.

Parker and Axtell (2001) suggested that the association between POS and OCB can be strengthened through perspective taking. Perspective taking refers to an individual’s attempt to understand the thoughts, motivations, and behaviors of others without subjective bias (Calvard et al., 2023). The concept here is that individuals will learn others’ ideas and thoughts through social activities that alter their prejudice and helps them accept or consider others’ perspectives. The decision-maker, as a consequence, will be able to consider and comprehend the performance indicators as well as the values and needs (Ng et al., 2022). Individuals with perspective taking are more likely to understand and accept the views of others, thereby stimulating personal altruistic motivation, and showing high OCB through voluntary service (Da’as, 2020). Base on this, POS can positively affect employees’ OCB performance through the interaction with volunteer participation motivation. It is believed that the above theory can be applied to the volunteering process of the frontliners of the National Immigration Agency, especially those who frequently interact with travelers entering or leaving Taiwan or those who counsel new immigrants. This is because by asking questions, listening carefully to others, and observing the behavior of others, employees will be able to pay more attention to the ideas and needs of others or accept the views of others. This change will enable frontliners to assist the public more effectively and efficiently, offer better public services, and improve the image of the government of Taiwan among domestic and foreign travelers. Furthermore, because employees’ positive motivation and perspective taking are a type of altruistic behavior of concern for others, more employee OCB will be triggered (Sabati, 2022). Although volunteer participation motivation is not entirely altruistic, the result may benefit other individuals (Stillman and Tonin, 2022).

Taken together, it is critical to enhance employees’ POS and trigger their volunteer participation motivation. Although both POS and volunteer participation motivation are related to employees’ personal perception and motivation, there are several ways for organizations to elicit their employees’ OCB. In fact, organizations may be able to influence their employees’ extra-role *via* multiple factors. For example, a manager can encourage his or her team members to accomplish a common goal of the organization by adopting the right leadership style. In fact, a manager’s leadership style has a decisive effect on how employees behave (Shofiyuddin et al., 2021). Studies have shown that by enhancing group identification and cohesion, an organization’s leader can trigger employees’ OCB by creating a positive organizational climate (Kao, 2017; Liao et al., 2022). Organizations can also use various formal and informal systems to create a good atmosphere and shape employees’ positive thinking and motivation for more OCB (Schmidthuber and Hilgers, 2019).

An organization can trigger employees’ OCB by enabling employees to perceive their contributions and welfare as valued by their organization and by providing various incentives. At the same time, the organization can work on employees’ volunteer participation motivation to use their desires to care for others in order to elicit more OCB. Moreover, transformational leadership and organizational climate not only encourage altruistic behavior but also reinforce employees’ POS and increase their volunteer participation motivation, and consequently, more OCB can be triggered. Although the above viewpoints have been confirmed by various studies (Astuty and Udin, 2020; Shofiyuddin et al., 2021), possible reinforcers for the relationship between POS and OCB have rarely been explored. This study considered employees’ motivation to volunteer an important factor catalyzing the relationship between POS and OCB, and for most volunteers, feedback mechanisms and altruistic motivations are the primary factors of volunteer participation motivation. Although various factors motivate people to volunteer, most researchers agree that caring for other people, which is an altruistic behavior, is one of these factors (e.g., Pan et al., 2022; Pfattheicher et al., 2022). For example, since 2012, the Taiwan Immigration agency has promoted the voluntary grouping of employees, using holidays to combine volunteer workers in the community to visit the homes of foreign immigrants in need of assistance, such as those living alone or those with limited mobility, to visit and provide necessary assistance. Such as assisting in medical treatment or helping to clean the home environment to encourage altruistic behavior of immigration officers and enhance the image of the organization. Such activities are called “going to the countryside service activities.” Based on the aforementioned, the association between POS and OCB can be reinforced by encouraging people to volunteer. Furthermore, this study has included organizational factors, i.e., transformational leadership and organizational climate, in exploring ways to encourage employees’ OCB.

According to the 2020 statistics database of the Tourism Bureau of Taiwan’s Ministry of Transportation (Tourism Bureau, Republic of China (Taiwan), 2020), before the COVID-19 outbreak, there were approximately 11.84 million tourists came to Taiwan in 2019, a record high and an increase of 7% from 11.07 million in 2018. According to the WTTC (2018) survey on Taiwan’s tourism employment environment, Taiwan’s tourism industry, including related industries, provided 264,000 job opportunities in 2017, accounting for 2.3% of the employment. Therefore, the number of employees in the industry or related industries has created a considerable proportion of Taiwan’s employment population, and its overall tourism output value accounts for about 2.47% of Taiwan’s GDP, which shows the importance of the tourism industry to Taiwan (WTTC, 2019). Although COVID-19 has caused a huge proportion of job losses to the global tourism industry, the epidemic is gradually fading and borders are being lifted. Under such circumstance, Taiwan not only needs to catch up quickly in the human resources of the tourism industry, but also the quality of human resources related to border control needs to be improved. Human resources are the core factor of organizational performance. Therefore, by exploring the topics of this research, it is especially important for Taiwan’s National Immigration Agency, which provides services to travelers entering or leaving Taiwan, new immigrants, and foreign workers. Because most existing OCB studies are focused on the antecedents or results of OCB, it is important to jointly explore the moderating effect of volunteer participation motivation and organizational factors. This can make up for the gap of existing research on how organizations can strengthen employees’ volunteer participation motivation, that is, encourage employees to “*perspective taking*” to enhance the relationship between organizational support and OCB.

Considering the level of analysis, because the transformational leadership of managers and organizational climate are organizational factors, they are group-level constructs. As a result, their effect on employees’ attitude or behavior should be analyzed and explored from a cross-level perspective. Therefore, this study views transformational leadership as a group-level variable. Besides evaluating individual-level variables, it is also critical to address the overall effect of a leader’s behavior on the group. Organizational climate was considered as an organization-level variable in this study. Employees’ perception about an organization’s climate can be pluralistic, and different behaviors are elicited in employees (Kao, 2017). Taken together, in this study, the frontliners of Taiwan’s National Immigration Agency were the study subjects, POS was the antecedent variable, volunteer participation motivation was the moderating variable, and OCB was the dependent variable. The objective of this study was to examine the relationship among the three variables above as well as the moderating effect of volunteer participation motivation. The cross-level effect of group variables, i.e., leadership style and organizational climate, on individual-level variables was also explored. In other words, we take this multi-level analytical approach to address knowledge gaps about the factors that affect the relationship between POS and OCB.

## 2. Conceptual framework

### 2.1. Concepts

An organization’s employees would have an overall perception of the organization based on whether their contributions and welfare are valued by the organization, which is perceived organizational support (Kurtessis et al., 2017). It can be defined as the belief that employees develop in their minds to assess how much the organization values their contributions and welfare. The social exchange theory suggests that employees who perceive that their contributions and welfare are valued by their organization (i.e., POS) will feel obligated to assist their organization in attaining its goal (Eisenberger et al., 1986), and because of this sense of obligation, these employees will exhibit not only in-role behavior but also extra-role behavior, such as OCB (Abdullah and Wider, 2022). Studies on volunteer participation motivation emerged in 2000, Wang (2001) proposed an idea of volunteers’ various motivations based on Clary and Snyder (1991) and Lucas and Williams (2000). These motivations include altruistic values, personal development, community concerns, ego enhancement, and social adjustment. Employee volunteer motivation can be defined as the motivation of employees to voluntarily participate in activities or affairs outside the organization, which is mainly based on social orientation and personal growth needs (Hurtz and Williams, 2009). Kim et al. (2015) pointed out that volunteer participation motivation originated from unsatisfied needs can be physiological or a drive. Overall, volunteer participation motivation is part of social motivation and has an altruistic nature (Han et al., 2020). As a result, volunteers are motivated to volunteer to care for other people. In addition, OCB is defined as an employee’s voluntary behavior, which is not restricted by the work contract and can effectively promote the performance of the organization (Bakhshi et al., 2011). OCB is an unconditional work behavior of employees (Cho and Kao, 2022). Although this type of behavior is not explicitly regulated in job descriptions, it is accepted by the organization (Qiu and Dooley, 2022). More importantly, OCB is a voluntary behavior of employees (Rizaie et al., 2023). Internally, OCB allows organizations to operate more effectively, thereby enhancing organizational performance (Williams and Polito, 2022). Externally, OCB improves service quality and customer satisfaction (Wang and Xiao, 2022). According to Azizaha et al. (2020) and Desky et al. (2020), OCB is a profound contribution that is beyond what is required by one’s job at the workplace and is rewarded by the organization based on the performed tasks. Vizano et al. (2020) revealed that employees’ OCB willingness is related to social exchange, and this theory is based on the hypothesis that there is a mutual and fair relationship between an organization and its employees. When employees have a positive perception of their organization, they will repay the organization practically and effectively, and OCB is the way that employees repay their organization (Shofiyuddin et al., 2021). OCB is a multidimensional behavior that is interpreted differently by researchers. Nevertheless, OCB overall is an interpersonal behavior beneficial to society, an explicit public welfare- and civic virtue-oriented behavior, and an in-role behavior about properly performing one’s duty (Kao, 2017). Since OCB is so important, organizations should understand what drives employees to exhibit more OCB. Studies have pointed out that the factors related to OCB include situational variables such as organizational justice, leadership support (Pletzer et al., 2021), and organizational climate (Kao, 2017). Job-related attitudes might include job satisfaction or organizational commitment (Shahjehan et al., 2019), individual differences, such as gender (Ng et al., 2016), cognitive ability (Miao et al., 2018), or caring altruistic behavior characteristics (Cho and Kao, 2022). For example, Thompson et al. (2020) revealed that women are more likely to feel obligated to show more extra-role behavior for the organization even when they perceive less organizational support. In addition, the relationship between POS and OCB can be either reinforced through factors such as person-organization fit (Jehanzeb, 2020) and psychological capital (Shaheen et al., 2016) or weakened by factors such as psychological contract breach (Islam et al., 2017). Interactions of these different classes of correlates are frequently used to predict employees’ OCB (Newman et al., 2017). Based on this, both organizational and personal factors are sufficient to influence the performance of employee OCB.

Transformational leadership can be defined as a process of organizational change that can combine the common needs and desires of organizational members. A leadership style based on members’ consensus on organizational commitment, where leaders create favorable conditions for personnel beliefs and behavior change (Benefiel et al., 2014). Studies have shown that a transformational leader encourages his/her subordinates to adopt a new perspective (e.g., intellectual stimulation) for problem solving. Moreover, a transformational leader should provide support and encouragement (e.g., individualized consideration), spreads a vision (e.g., inspirational motivation) and elicits both affection and identification (e.g., charisma) (Gabriel et al., 2022). Like a mentor or a coach, a transformational leader listens attentively to his or her subordinates, cares for each employee individually, and pays attention to the achievement and the improvement of subordinates. Furthermore, a transformational leader encourages subordinates to take more responsibility, thereby helping them reach their full potential (Chebon et al., 2019; Bin Bakr and Alfayez, 2021). A distinctive characteristic of transformational leaders is that they share a group interest-oriented vision with the people around them (Fareed and Su, 2022). In addition, a transformational leader tends to create a committed work climate. They empower their followers and provide them with enough support for innovation at work (Astuty and Udin, 2020). Based on this, transformational leadership increases employees’ OCB by boosting their POS and triggering their volunteer participation motivation (Dinc et al., 2022; Du and Yan, 2022; Guarana and Avolio, 2022). As for the climate, it is a way for people to learn about their work environment. It represents the perception model or theme experienced by employees. It is also about organization members conceptualizing all their workplace related experiences (Hsieh and Kao, 2022; Sawyer, 2022; Shenk and Gutowski, 2022). Litwin and Stringer (1974) defined organizational climate as an assessable work environmental feature, and it is an individual’s direct or indirect perception about his or her life and work in the work environment. Therefore, this study confirms that organizational climate can be defined as “the employee’s awareness of some events, activities and procedures in a certain environment, as well as those behaviors that may be rewarded, supported, and expected,” that is, it can be described as the shared cognition of members of the same organization. Organizational climate is assumed to affect employees’ motivation and behavior (Kataria et al., 2022). This is because organizational climate affects how an individual perceives his or her daily business in the organization (Kao, 2017). Moreover, through engagement and experience, employees’ behavior is affected by the internal environment of their organization (Hoang, 2022). As a result, a positive organizational climate not only encourages job performance and a positive organization-employee relationship, but also improves employee job satisfaction that is capable of triggering employees’ volunteer participation motivation (Gheitani et al., 2018; Saks, 2022). Group cohesion and socialization experiences formed by organizational climate can affect employees’ willingness to increase their extra-role behavior (Schmidthuber and Hilgers, 2019), such as doing more volunteer work or engaging themselves more in civic activities good for their organization (Soelton et al., 2020).

In summary, POS can promote employees’ OCB display, and organizations can encourage employees to display more OCB by encouraging employees to participate in volunteer activities. At the same time, through the leadership style of the supervisor and the establishment of a good organizational climate, the organization can strengthen the POS of employees and the participation motivation of volunteers, so to induce them to show civic behavior that is beneficial to the organization.

### 2.2. Hypothesis development

#### 2.2.1. Relationship between perceived organizational support and organizational citizenship behavior

Hayat Bhatti et al. (2019) believed that in the foundation of trust, employees have the willingness to demonstrate OCB because they believe that the organization or managers will have peer feedback after their efforts. The Qi et al.’s (2019) study pointed out that based on the exchange norm of reciprocity, when employees perceive organizational support, they develop a sense of obligation to help the organization achieve its goals. Because from the perspective of social exchange theory, when employees feel the help of the organization, they will give back to the organization with harder hard work and loyalty, or help employees in difficulty. Perceived organizational support comes from how employees attribute and judge how the organization treats them (Silva et al., 2022). Therefore, when members who join an organization feel considered as part of the organization and are highly valued, employees will feel that they have a responsibility to contribute adequately to the organization to provide the best performance for the organization (Kristiani et al., 2019).

Based on the above, studies have shown that based on the reciprocal norm, employees who perceive support from their organization tend to feel obligated to help their organization achieve its goal (Thompson et al., 2020). According to the social exchange theory, employees who perceive organizational support will work harder and be more loyal to the organization in return (Imran et al., 2020). Therefore, the employees of an organization who perceive themselves as being treated as members of their organization and valued by their organization will consider themselves responsible for making as many contributions as possible to their organization; so the organization can perform its best (Ridwan et al., 2020). In additional, the psychological mechanism of employees’ perception of their organization’s loyalty (commitment) is related to the concept of social exchange and a psychological contract between employees and their organization (Garcia et al., 2021). POS is capable of affecting employees’ work attitude and behavior through the effort-anticipated reward association and the reinforcement of personal social emotional need (Shabbir et al., 2021). Consequently, organizations can use employees’ POS to boost their OCB. Moreover, according to the social exchange theory and the reciprocal norm, employees develop a general belief assessing the extent to which their organization values their contributions and wellbeing. Employees perceiving a trustworthy relationship with their organization are likely to demonstrate OCB (Kurtessis et al., 2017; Aboramadan et al., 2022).

According to the above ideas, this study proposed the first hypothesis:

**H1: Perceived organizational support has a positive effect on organizational citizenship behavior.**

#### 2.2.2. Volunteer participation motivation has a moderating effect on the relationship between POS and OCB

Han et al. (2019) suggested that by offering incentives, organizations can elicit employees’ OCB. Therefore, when an organization cares about its employees’ wellbeing, values their contributions, and provides favorable work conditions, its employees will perceive organizational support and appreciate the organization. These employees will also show a strong sense of obligation, which prompts them to exhibit OCB in return (Thompson et al., 2020). Parker and Axtell (2001) proposed the perspective taking theory and suggested that the effect of taking the perspective of other people would change an employee’s original traits and dispositions, and the magnitude of the change depends upon the person’s motivation (Škerlavaj et al., 2018). Within an individual’s scope of work, the social motivation of volunteering directly benefits other people while improving one’s work performance and the drive to succeed, and therefore, it is OCB-related (Robison, 2022). Moreover, people with perspective taking can put themselves in others’ position to understand and feel the thoughts and ideas of others, so as to form a motivation to help others and promote them to show more altruistic behaviors, such as OCB (Sabati, 2022). Therefore, in the influence of POS on OCB, if the voluntary service with altruistic motives of volunteers can be used as perspective taking, the organization’s support for them can be better understood, which will eventually increase OCB (Kim et al., 2019). Furthermore, studies have shown that employees showing a stronger volunteer participation motivation are likely to be more engaged at work and more satisfied with their work (Dal Corso et al., 2019). In terms of engagement motivations, the key motivations are interpersonal interaction, serving others, knowledge acquisition, and keeping in touch with society (Arai, 2000). Researchers have discovered that volunteering is an engagement behavior based on social responsibility; it is a virtue of helping other people and an organizational altruistic behavior (Ficapal-Cusí et al., 2020).

Taken together, employees who perceive themselves as well-treated by their organization will in return demonstrate citizenship behavior beneficial to their organization. This mentality of reciprocation or exchange will affect employees’ attitude toward their organization and reflect on their work attitude or behavior. Therefore, POS and volunteer participation motivation will affect OCB *via* different social exchange routes, and employees’ volunteer activities have a positive catalytic effect on the relationship between POS and OCB. In other words, POS can positively affect employees’ OCB performance through the interaction with volunteer participation motivation.

According to the ideas above, the second hypothesis was proposed as follows:

**H2: Volunteer participation motivation has a moderating effect on the relationship between POS and OCB.**

#### 2.2.3. Relationship between transformational leadership and organizational climate

The leader of an organization plays a key role in affecting the work environment and employees’ perception of work (Pancasila et al., 2020). Avolio et al. (1999) pointed out that transformational leaders would apply their influence to integrate leader-subordinate interactions. At the same time, they would offer personalized care, stimulate employees intellectually, and establish an interactive and creative process between leaders and their followers through motivation and spiritual inspiration. By doing so, they would encourage employees, win their admiration, respect and honesty, and aggregate the power from their followers, which together will promote group cohesion (Jiang and Chen, 2018). Some studies have shown that transformational leaders with a good leadership style can boost employees’ confidence in other team members, and employees will believe that their team members will offer help when they are in need. This will therefore improve team cohesiveness (Asgari et al., 2020). According to the above findings, transformational leadership cultivates group identification and cohesion, links team members together, and consequently affects organizational climate (Mach et al., 2022).

As a result, the third hypothesis was proposed:

**H3: Transformational leadership has a positive effect on organizational climate.**

#### 2.2.4. The cross-level effect of transformational leadership on POS and volunteer participation motivation

Research has shown that transformational leaders can inspire those around them to emphasize a vision of group interests (Veríssimo and Lacerda, 2015), create a committed work climate, empower their followers, and provide sufficient support to achieve innovation at work (Astuty and Udin, 2020). In addition, a leader with a transformational leadership style tends to motivate his/her subordinates to exceed their expected output by changing their vision, being role models, providing support and inspiring their desire to change for the better (Suifan et al., 2018). Furthermore, a study has shown that transformational leadership encourages people to value a common interest-based vision (Guarana and Avolio, 2022). It also creates a work climate for organizational identification, supports and encourages subordinates, and spreads the organization’s vision thereby making employees feel emotionally connected to the organization and identify themselves with the organization (Dinc et al., 2022; Du and Yan, 2022). At the same time, employees are encouraged to bear more responsibility for the development of positive motivation and altruistic behavior (Astuty and Udin, 2020). Based on the previous mentions, transformational leadership styles will have a great impact on how subordinates act (Shofiyuddin et al., 2021), such as improving POS and showing more OCB (Bacha and Kosa, 2022).

Managers and other leaders in an organization have a critical influence on organizational incentives. For example, transformational leaders can timely provide employees with the resources they need through the role of leadership, such as playing the role of monitor or coach (Chebon et al., 2019; Bin Bakr and Alfayez, 2021). Therefore, employees’ POS has been viewed to be primarily associated with their managers (Lipponen et al., 2018). The above functions of a leader make employees feel being strongly supported by management, a way of showing them that their organization cares about them. In this case, the employees will perceive support from their organization (Kurtessis et al., 2017). In fact, some recent studies have shown that transformational leadership facilitates employees’ perception of support from the organization. For example, Asgari et al. (2020) studied college students and proposed that transformational leadership has a positive effect on employees’ POS. Hermawati et al. (2021) studied perceived organization support and demonstrated that transformational leadership has an effect on the followers’ POS. Dinc et al. (2022) examined the effect of transformational leadership on employees’ creativity and found that transformational leadership plays a critical role in employees’ POS. In multi-level organization theory models, group-level variables affect individual-level outcome variables cross-levels (Raudenbush and Bryk, 2002). In other words, transformational leadership at the group level not only affects organizational climate, but also affects individual POS and volunteer participation motivation cross-levels as well. Based on this, research on leadership must pay attention to the correlation between variables at different levels. The above theoretical discussion or research shows that transformational leadership can affect the outcome variables of employees’ POS and volunteer participation motivation, and can influence the context variables of the organization (Elgamal, 2004). Therefore, through multi-level research, employees’ perceptions of leaders can be aggregated to the group level, and their relationship with POS and volunteer participation motivation can be tested.

As a result, the fourth hypothesis was proposed:

**H4: Transformational leadership has a positive cross-level effect on POS.**

Transformational leadership makes employees’ job more meaningful, creates high-quality teamwork, and give employees a feeling of satisfaction. Transformational leadership has a huge effect on maintaining the environment driving volunteering (Althnayan et al., 2022). Individuals who see their job as meaningful or have an autonomous motivation are more likely to feel satisfied with their job and to help other people (Purvanova et al., 2006). Volunteering is a way to show one’s altruistic value, to establish a strong relationship, to improve one’s self-esteem, to shift one’s attention away from personal issues, to learn and acquire new knowledge and skills about the world, and to improve career prospects (Afkhami et al., 2019). Transformational leadership affects employees’ volunteer participation motivation by satisfying their needs for interpersonal relationship and building good relationships (Chan, 2020). Moreover, transformational leaders link work to employees’ values through their charismatic leadership style, boost employees’ confidence, and facilitate team identification and cohesion, which together elicit employees’ volunteer participation motivation (Shofiyuddin et al., 2021).

As a result, the fifth hypothesis was proposed:

**H5: Transformational leadership has a positive effect on volunteer participation motivation.**

#### 2.2.5. Organizational climate’s cross-level effect on volunteer participation motivation and OCB

Employees constitute an important part of every organization, and form a general perception of the organization based on their work in the organization and their experience with the organization’s internal environment such as organization management, organizational culture, and the content and place of work. The behavior of employees is affected by this general perception (Licciardello et al., 2013). A positive organizational climate enhances employees’ job performance, creates a positive employee relationship, and boosts their job satisfaction. Therefore, a positive work environment through good interpersonal relationships and a healthy organizational climate can help motivate people to volunteer (Lee and Brudney, 2015). Suresh and Venkatammal (2010) believed that organizational climate is a collection of attitudes that an organization influences individuals and groups, like rewards and interpersonal relationships. Gholami et al. (2015) viewed organizational climate as employees’ perceptions of organizational characteristics, such as leadership styles, decision-making processes, and work norms. Furthermore, according to Sudibjo and Nasution (2020), and Suprapti et al. (2020), the benefits of the work environment are to create of passion for work, and improve work performance. Therefore, a positive work environment can create a good organizational climate, promote the sharing of the organization’s values, beliefs, and behaviors, and then induce employees to exhibit more behaviors that are beneficial to the organization (Novitasari and Iskandar, 2022).

Many studies have found that fairness (Salam, 2020), a sense of self efficacy (Zhou and Qian, 2021), collective efficacy (Strydom, 2021), and group cohesion (Schmidthuber and Hilgers, 2019) are the antecedents of employees’ ex-role behavior. Engagement in volunteer services or activities gives people an opportunity to show their altruistic values (Clary et al., 1998) and trigger their ex-role behavior. For example, Azizaha et al. (2020) and Desky et al. (2020) pointed out that the motive of an individual, group cohesion and employees’ attitude are closely related to triggering employees’ OCB. Therefore, establishing a good organizational climate and as such a strong group cohesion will further encourage employees’ OCB (Schmidthuber and Hilgers, 2019). This study defines organizational climate as a group-level variable. Since this study uses cross-level analysis, hypotheses about the group-level effect on individual-level dependent variables can be tested (Cho and Kao, 2022). In summary, organizational climate can affect volunteer participation motivation and organizational outcomes of OCB.

Consequently, the sixth and seventh hypotheses were proposed:

**H6: Organizational climate has a positive cross-level effect on volunteer participation motivation.**

**H7: Organizational climate has a positive cross-level effect on OCB.**

## 3. Research methodology

### 3.1. Research structure

**FIGURE 1:**
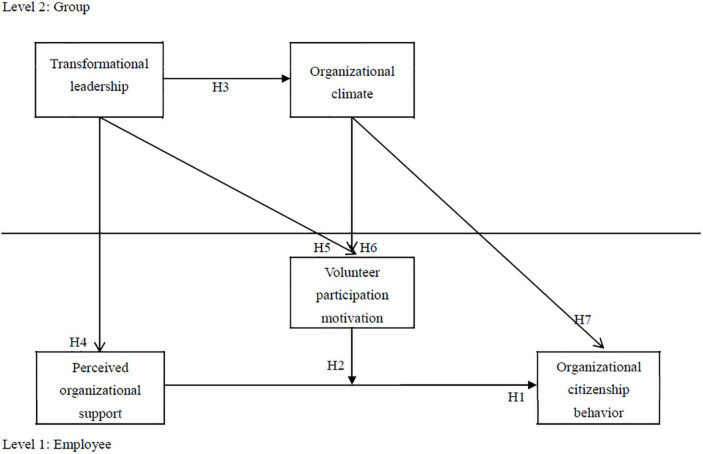
Research model.

The research structure is presented in Figure 1. Based on the research objectives and hypotheses, the following effects and relationships were tested:

1) Causation between individual-level variables.
2) The moderating effect of volunteer participation motivation on individual-level variables.
3) Causation between group-level variables.
4) The cross-level effect of group-level variables.

### 3.2. Research subjects, sampling and strategies for research, or data collection

This study adopts a quantitative methodology and retrieves the data by questionnaires. In addition, this study was approved by the Research Ethic Committee of a university in Taiwan. This study sampled frontliners of Taiwan’s National Immigration Agency to be the study subjects, and because of cross-level issues, this study had to take sampling from different levels into consideration. To ensure data quality and obtain reliable group-level data, this study made reference to the cluster sampling approach used in Kao (2017), and when selecting divisions for sampling the study subjects, this study set the following sampling criteria: (1) Each division should have at least 10 employees (excluding the director and the deputy director); (2) the respondents should have worked at their division for more than 6 months to ensure that the respondents have a concrete understanding of the study variables. As for the sampling method, because of limited manpower and financial resources, when collecting data, stratified sampling was conducted first, followed by quota sampling. In this study, there were two types of interviewees; one consisted of frontliners of the Border Affairs Corps under the National Immigration Agency, Taiwan. These people are responsible for checking the passport of travelers entering or exiting Taiwan at international airports or ports. They are also in charge of security-related issues at the border and for interviewing foreigners working in Taiwan or coming to Taiwan for marriage to a Taiwanese. The other type of interviewees were frontliners of the Service Affairs Corps at various Service Stations across Taiwan. They are responsible for Taiwanese with household registration entering or exiting Taiwan, reviewing and approving the stay, temporary residence, or permanent residence of travelers from mainland China, Hong Kong, Mao Cao, and other countries worldwide, and for offering immigration counseling services. In terms of the research questionnaires, two questionnaire types were used in this study. The first type was for frontliners of the sampled divisions (non-management), and the second type was for management, i.e., the direct managers, of the divisions that have employees sampled for the first questionnaire. The objective here was to determine any discrepancy between employees’ self-perceived OCB and their OCB as perceived by their managers.

In addition, in order to make the sample to effectively represent the population, this study adopts a stratified two-stage sampling design, and is based on the principle of probability proportional to size (PPS) sampling. According to the current organizational structure of the Taiwan Immigration Agency, a total of 9 service centers of the Northern, Central and Southern Taiwan Administration Corps were selected, and 8 brigades (4 brigades each at the airport and 4 at the port) were selected from the 16 brigades under the Border Affairs Corps according to their regions and work attributes to maintain the principle that the odds of being selected for each case are roughly the same. In addition, before the questionnaire was administered, the respondents were explained the research themes, methods, and how to use future research results, and informed their basic rights in the research, such as the confidentiality of the respondents’ relevant information. The respondents were told to be freely to participate in research and have the right to withdraw from research at any time to meet the research ethics requirements of “informed consent.” Based on the sampling procedures and methods, a total of 289 valid questionnaires were obtained in this study, and the specific information will be described later.

### 3.3. Research questionnaires

This study’s questionnaires are mostly based on questionnaires presented in studies by European and American researchers, and when translating these works, the researcher of this study followed the suggestion from Brislin (1986). To ensure the accuracy of the translation, the works were translated by bilingual translators, and reverse translation of the research variables was also performed to make sure that the Chinese version maintains the concepts of the original questionnaires. A 5-point Likert Scale ranging from strongly agree (5 points) to strongly disagree (1 point) was adopted. The questionnaires are explained below.

#### 3.3.1. Perceived organizational support

Perceived organizational support is the development of an organization’s beliefs in its employees and when employees recognize that their contribution and well-being are highly valued by the organization, employees will feel obligated to help their organization achieve its goals. Therefore, it is very important for the organization to build this belief among employees. This questionnaire was modified from the one used by Eisenberger et al. (1997). There were 15 questions after the modification, and the objective was to measure the elements of POS. One of the questions was “Does the organization value my contributions?” Another question in the questionnaire was “Does the organization actively provide administrative or training resources?”

#### 3.3.2. Volunteer participation motivation

Volunteer participation motivation refers to the motivation of employees to voluntarily participate in activities or affairs outside the organization. It is part of social motivation and has altruism, which makes volunteers motivated to care for others voluntarily. This study adopted and modified the six aspects of volunteer participation motivation of Wan et al. (2007). In total, there were 32 questions in the revised questionnaire, and they were used to assess the six elements of volunteer participation motivation. There were ten questions for enriching life and learning motivation, the first element. One of the questions was “does volunteering enrich my life?” There were five questions for the element “social responsibility,” and one of the questions was “can I make contributions to society by volunteering?” There were five questions for self-development motivation, and one of the questions was “does volunteering provide self-development opportunities?” There were four questions for the element “motivation for achievement,” and one of the questions was “can I serve the public by volunteering?” For the element “institutional characteristics,” there were four questions, and one of the questions was “By volunteering can I obtain printed materials from that organization or can I participate in activities held by the organization for free or with a discount?” The last element was “will realization motivation,” and there were four questions, one of which was “can I volunteer to reach my full potential?”

#### 3.3.3. Organizational citizenship behavior

Organizational citizenship behavior is considered as an unconditional work behavior of employees. Although this behavior is not clearly stipulated in the job description, employees still voluntarily engage in it, and the display of this behavior can effectively promote the performance of the organization. This questionnaire was developed based on Farh et al. (2004) and Lin (2007). Three OCB dimensions were included in this inventory: in-role behavior, organizational charitable behavior, and interpersonal altruistic behavior. Each dimension contained two sub-dimensions. For in-role behavior, the sub-dimensions were identification with the company and taking initiative. For organizational charitable behavior, the sub-dimensions were diligence, prudence and protecting the company’s resources. For interpersonal altruistic behavior, the sub-dimensions were interpersonal harmony and assisting one’s colleagues. When conducting the survey, both the employees and their managers were asked to fill out the questionnaires in order to eliminate the possibility of common method variance. By doing so, the researcher could assess if there is any discrepancy between the actual perception of the employees and the employees’ behavioral evaluation conducted by their managers. Both questionnaires contained six questions on identification with the company. One question from that part was “should I (the subordinate) try my best to protect the company’s image and actively participate in all related activities?” There were three questions under taking initiative and one of them was “If it is required for business, should I (the subordinate) go to the office earlier to do my work?” For diligence and prudence, there were 11 questions. One of the questions was “should I (the subordinate) put effort into self-enrichment in order to improve the quality of my work?” For saving the company’s resources, there were three questions. One of the questions was “should I (the subordinate) try to save organizational resources such as water, electricity, and office stationery?” For interpersonal harmony, there were five questions. One of the questions was “should I (the subordinate) actively communicate and coordinate with my colleagues?” There were three questions under assisting colleagues. One of the questions was “should I (the subordinate) be happy to assist my colleagues and to solve problems encountered at work?” Altogether, there were 31 questions and six sub-dimensions.

#### 3.3.4. Transformational leadership

Transformational leadership is a process of organizational change, based on members’ consensus on organizational commitment, it can combine the common needs and aspirations of organizational members, share a group-interest-oriented vision with those around it, and create a loyal working climate. To evaluate transformational leadership, this study adopted the multifactor leadership questionnaire (MLQ) developed by Bass and Avolio (1997). MLQ covers four dimensions: six questions on idealized influence, five questions on inspirational motivation, four questions on intellectual stimulation, and four questions on individualized consideration. For idealized influence, it was used to measure the role of the leader in his or her job position. One of the questions from the idealized influence dimension is “would my supervisor put group interest above his own interest?” One of the questions from the inspirational motivation dimension was “does my supervisor have a positive view of the future?” For the intellectual stimulation dimension, one of the questions was “Why does my supervisor ask me to see a problem from various aspects?” Last, for the individualized consideration dimension, one of the questions was “does my supervisor help me realize my potentials?”

#### 3.3.5. Organizational climate

Organizational climate is an evaluable feature of the work environment, which is the direct or indirect perception of an individual’s life and job in the work environment, and it is the common understanding among members of the same organization. This study revised the organizational climate questionnaire used in Kao (2017), which is based on the Litwin and Stringer Organizational Climate Questionnaire (LSOCQ) (Litwin and Stringer, 1974). The organizational climate questionnaire used here has four dimensions and 18 questions in total: six questions on management style, four questions on interpersonal relationship, four questions on structural climate, and four questions on climate responsibility. The organizational climate questionnaire was used to evaluate management styles (e.g., the job promotion system), interpersonal relationships (e.g., a friendly climate), structures (e.g., a very high performance standard), and responsibility (e.g., individuals do not need to take any responsibility).

#### 3.3.6. Control variables

According to studies such as Farh et al. (2004), Kao (2017), and Cho and Kao (2022), this study included age, education, seniority as the control variables and examined their relationships with other study variables.

## 4. Results

### 4.1. Background information

According to the sampling procedure, this study selected a total of 9 service centers from the Taiwan Immigration Administration Corps, and 8 brigades from the Border Affairs Corps (4 brigades each from the airport and port). There were more than 10 general employees and at least 2 managers who were selected. During the test, the time between the rotations of each unit were used to test the respondents who have finished the service until all those who work on that day were tested. A total of 289 employee questionnaires were completed and returned; 227 of them were from male employees, and 62 from female employees. This gender ratio was similar to that of employees in the National Immigration Agency. The number of respondents accounted for about 20.81% (289/1389) of the population. Most of the employees had a bachelor’s degree, and 28.5% of them had a diploma from Taiwan Police College. The average age of the interviewees was 39.92, and they had worked for 11.32 years. They had worked at the current division for 4.37 years, and in each division there was an average of 21.58 people. Thirty-two participants had completed the manager questionnaire; 24 were male and 8 were female. Most of the respondents had a postgraduate degree (59.38%), while 37.5% of them had a diploma from Taiwan Center Police University. The average age of the respondents was 45.36, and they had worked for 15.43 years. At the current division, the respondents had worked for 3.21 years. In order to determine differences in the perception of employees’ OCB between frontliners and their managers, this study performed a *t*-test and no significant difference was found. Therefore, both questionnaires were merged for hypothesis testing.

Table 1 presents the mean, standard deviation, correlation coefficient, and α of the study variables. According to Table 1, the control variables had no significant effect on other variables. The study variables had a good reliability (0.7 or above), and were positively correlated. In this study, the researcher used LISREL (maximum likelihood estimation) for confirmatory factor analysis (CFA) to determine if POS, volunteer participation motivation, OCB, transformational leadership, and organizational climate are different constructs. The results are presented in Table 2, which shows that the five study variables were distinct constructs. In addition, SPSS Windows 22 was used to analyze the cross-level effect.

**TABLE 1:** Descriptive statistics, correlation coefficient, and alpha coefficient.

	*M*	*SD*	α coefficient	Research variables				
				**(1)**	**(2)**	**(3)**	**(4)**	**(5)**
(1) Perceived organizational support	3.542	0.799	0.881	1				
(2) Volunteer participation motivation	3.128	0.623	0.702	0.328***	1			
(3) OCB	3.689	0.565	0.801	0.241*	0.401***	1		
(4) Transformational leadership	3.427	0.299	0.888	0.179*	0.212*	0.254*	1	
(5) Organizational climate	3.579	0.285	0.812	0.194*	0.293**	0.377***	0.321***	1

(1)–(3), individual-level research variables; (4)–(5), group-level research variables.

*p < 0. 05; **p < 0.01; ***p < 0.001.

**TABLE 2:** Goodness of fit indicators for individual-level variables.

Research variable	χ2/df		GFI		NNFI		PGFI		RMSEA	
	**Observed value**	**Ideal value**	**Observed value**	**Ideal value**	**Observed value**	**Ideal value**	**Observed value**	**Ideal value**	**Observed value**	**Ideal value**
Transformational leadership	2.42		0.94		0.94		0.67		0.041	
Organizational climate	2.25		0.94		0.95		0.71		0.036	
Perceived organizational support	2.61	1.00∼3.00	0.93	>0.9	0.93	>0.9	0.63	≥0.5	0.045	≤0.05
Volunteer participation motivation	2.87		0.92		0.93		0.55		0.048	
OCB	1.89		0.96		0.97		0.79		0.027	
References	Schumacker and Lomax (1996)		Bentler (1990)		Bagozzi and Yi (1988)				Browne and Cudeck (1993)	

### 4.2. Testing aggregated data

This study tested the group effect of transformational leadership and organizational climate (*F*-value) to determine if data can be aggregated for group-level analysis. The following parameters were used during data analysis, for transformational leadership, η2 = 0.472, *F* = 7.02, and *p* < 0.001; and for organizational climate, η2 = 0.319, *F* = 4.02, and *p* < 0.001. Therefore, the group effect of transformational leadership and organizational climate was statistically significant. In addition, to assess the consistency of rating by group members, James (1982) suggested the use of intraclass correlations (ICCs). ICC (1) reflects the consistency of rating by members on the same team. The ICC (1) coefficient criteria ranged from 0.05 to 0.3 (Bliese, 2000). In this study, the ICC (1) coefficient was 0.17 for transformational leadership and 0.22 for organizational climate. Therefore, the ICC (1) of the group variables was statistically significant. To demonstrate the appropriateness of the aggregation, this study also calculated the r_wg_ of transformational leadership and organizational climate. The mean r_wg_ of transformational leadership and organizational climate was 0.75 and 0.79, respectively, both satisfying the critical value of 0.70 (James et al., 1984). The statistical data of aggregation suggested that the individual-level data can be aggregated for group-level analysis.

### 4.3. Hypothesis testing

#### 4.3.1. Hypothesis testing: Individual-level and group-level variables

This study tested the relationship between the control variables, the individual-level variables, and the group-level variables by Hierarchical Routing Architecture (HRA). Model 1 in Table 3 shows that group-level control variables had no significant effect on organizational climate. Model 2 shows that transformational leadership had a significant effect on organizational climate (β = 0.307, *p* < 0.001), and after adjustment, it had a *R*^2^ of 0.287. The *F*-value was statistically significant (*p* < 0.001). Models 3 and 5 show that individual-level control variables had no significant effect on either volunteer participation motivation or OCB. In addition, Model 4 shows that POS had a significantly positive effect on volunteer participation motivation (β = 0.319, *p* < 0.001). The adjusted R^2^ was 0.273, and the *F*-value was statistically significant (*p* < 0.001). It can be seen from Model 7 that volunteer participation motivation had a moderating effect on the relationship between POS and OCB (β = 0.335, *p* < 0.05). Therefore, H1 to H3 cannot be rejected. In other words, POS had a significantly positive effect on volunteer participation motivation, while volunteer participation motivation had a moderating effect on the relationship between POS and OCB. Transformational leadership had a significantly positive effect on organizational climate.

**TABLE 3:** Hierarchical regression analysis.

Model independent variables (group-level)	Model number						
	**1**	**2**	**3**	**4**	**5**	**6**	**7**
Group size (control variables)	0.024	0.008					
TL (independent variable)		0.307***					
*F*	0.192	20.124***					
Adj. *R*^2^	0.008	0.287					
**Model independent variables (individual-level)**							
Age			0.025	0.082	0.041	0.079	0.082
Education level			0.038	0.056	0.032	0.051	0.056
Years of service			0.049	0.066	0.058	0.084	0.066
POS				0.319***			
VPM							
OCB						0.239***	0.267***
POS × VPM							0.335***
*F*			0.804	24.392***	1.057	18.341***	36.458***
Adj. *R*^2^			0.002	0.312	0.018	0.273	0.407

Dependent variable: Model 1 and 2 are for OCL; model 3 and 4 for VPM; model 5, 6, and 7 for OCB.

TL, transformational leadership; OCL, organizational climate; POS, perceived organizational support; VPM: volunteer participation motivation (VPM). ***p < 0.001.

#### 4.3.2. Cross-level hypothesis testing

This study used the hierarchical linear model (HLM) to test the cross-level effect of group-level variables.

##### 4.3.2.1. The null mode

A HML null model with no explanatory variable was first set up to test whether the relationships among group variables, individual variables, and employees’ OCB were significant and to determine whether there was any significant difference between the interviewed divisions. Table 4 shows that the between-group variances were significantly non-zero (τ00 = 0.102, df = 24, Wald *Z* = 3.572, *p* < 0.01), and therefore employees’ OCB was different between divisions.

**TABLE 4:** Hierarchical Linear Modeling Results for Individual variables.

Variable	γ _01_	τ _00_		γ _11_
1. The null model		0.102***		
2. Context effects (intercepts-as-outcomes model)			(3) Organizational climate– Volunteer participation motivation	0.267** **(0.101)**
(1) Transformational leadership– Perceived organizational support	0.184* **(0.062)**		(4) Organizational climate–OCB	0.352*** **(0.092)**
(2) Transformational leadership– Volunteer participation motivation	0.201** **(0.154)**			

The numbers in bracket are standard error; (1) to (4) are the contextual effects of group-level variables on individual-level variables. For example, transformational leadership–perceived organizational support is the contextual effect of group-level transformational leadership on the individual-level perceived organizational support.

The table lists the indicators for tested hypotheses only.

The bold values indicate the test indicators for each hypothesis.

*p < 0.05; **p < 0.01; ***p < 0.001.

##### 4.3.2.2. Context effect

This study performed the HLM intercepts-as-outcome model testing on volunteer participation motivation and OCB, and the objective was to explain information related to Level 1 intercept variances. At the same time, this study used group-level transformational leadership and organizational climate as the explanatory variables of Level 2 and hypothesized that transformational leadership and organizational climate could positively and in a cross-level fashion affect POS, volunteer participation motivation, and OCB. To test the context effect of the group level on individual-level variables, this study used the γ_01_ parameters for the testing. Table 4 shows that transformational leadership had a cross-level main effect on POS (γ_01_ = 0.184, SE = 0.062, *t* = 2.01, *p* < 0.05) and on volunteer participation motivation (γ_01_ = 0.201, SE = 0.154, *t* = 2.87, *p* < 0.01); so did organizational climate on volunteer participation motivation (γ_01_ = 0.267, SE = 0.101, *t* = 3.16, *p* < 0.01) and on OCB (γ_01_ = 0.352, SE = 0.092, *t* = 3.88, *p* < 0.001). Therefore, H4, H5, H6, and H7 cannot be rejected.

## 5. Discussion and conclusion

### 5.1. Conclusion

The objective of this study was to explore the relationships between POS, volunteer participation motivation, and OCB in National Immigration Agency frontliners. This study also examined the moderating effect of volunteer participation motivation and the cross-level effect of transformational leadership and organizational climate. The results showed that all the hypotheses of this study were supported. It was found that employees’ POS had a positive effect on OCB, while volunteer participation motivation had a moderating effect on the relationships between the above mentioned variables. Furthermore, transformational leadership and organizational climate were found to have a cross-level effect on enhancing employees’ POS, boosting their motivation to volunteer, and triggering more OCB in employees.

In addition, this study has demonstrated that improving employees’ POS and triggering their volunteer participation motivation are important for increasing OCB in employees. This study also verified that an organization can trigger more employee OCB through the use of transformational leadership, which motivates members to achieve a common organizational goal, promote a vision encouraging people in the organization to value the group interest, create a work climate facilitating organization identification, and enhance employees’ group identification and cohesion. The above strategies enable a leader to optimize employees’ POS and motivate employees to volunteer, thereby inducing more OCB in the employees.

### 5.2. Management implications

This study used HLM to analyze and explain the cross-level effect of organizations. Appropriate statistical methods were used to assess the contextual effect of group-level variables on individual-level variables, so that the organizational or group effect on individual behavior can be concretely assessed. Secondly, the cross-level testing methods enabled the study to better understand the approaches and means, such as transformational leadership, that an organization can use to encourage its employees at the multidimensional organizational level to exhibit attitudes or behaviors benefiting the organization.

This study has made several important contributions to practice. First, the study result showed that a manager’s transformational leadership and organizational climate are important for frontliners of the organization. In a multi-level and service-oriented public sector, a manager’s leadership style and workplace climate play a critical role in shaping employees’ altruistic behavior and triggering various attitudes or behaviors of employees that are good for the organization. Secondly, this study found that a transformational leader encourages his or her subordinates to handle problems or take challenges by a new approach; they also enlighten the subordinates intellectually at work. Transformational leaders can handle not only the tasks assigned by superiors, but also deal with issues of “people” (i.e., subordinates) (Mahmood et al., 2018). Based on this, we advocate that the Taiwan Immigration Agency should train the direct supervisors of their front-line employees to have a transformational leadership style, so that these supervisors can organize his team, build a common understanding of employees’ goals or vision, and achieve organizational and personal goals through effective leadership, communication coordinate and cooperate to achieve organizational and personal goals, and show more OCB to improve organizational performance. Third, transformational leaders create a lively, enthusiastic, and pleasant climate in the organization, and guide employees to do their best to complete a task. Fourth, this study found that employees’ volunteer participation motivation can indeed reinforce a positive relationship between POS and OCB. Moreover, employees can also acquire valuable experiences from volunteering, which may encourage them to offer their organization concrete and constructive suggestions to improve their services to the public. Based on this, it is the responsibility of the supervisor to lead the department to establish a “correct” organizational clime. Therefore, this study believes that when recruiting employees, Taiwan’s immigration agency should focus on employees who can complement the organization, and provide opportunities for internal promotion in each position, so that employees can develop a sense of trust in the organization. In addition, the organization should establish a fair and immediate reward mechanism, based on the performance of the team, so that team members can establish a higher team awareness and are willing to work hard for the team’s common goals. Furthermore, the organization can also enhance employees’ awareness of teamwork through education and training to enhance organizational cohesion and promote organizational effectiveness (Para-González et al., 2018).

### 5.3. Research limitations and suggestions for future researchers

This study has obtained many findings. However, there are some limitations in research. Firstly, since this research is limited by the researchers’ time, manpower, and financial factors, the empirical objects are limited to the front-line employees and their managers who worked in the Taiwan Immigration Agency at the time of the questionnaire test, so whether the results of this study can be generalized to other similar agencies, such as border police, yet to be further analyzed and clarified. Therefore, we recommend that future researchers expand the scope of research to all border enforcers such as customs, police and coast guards. In addition, this research is mainly based on quantification, it is cross-sectional research. Therefore, this study suggests that subsequent researchers should add a longitudinal survey method to specifically measure the changes in respondents’ attitudes or behaviors in the research variants.

## Data availability statement

The original contributions presented in this study are included in this article/supplementary material, further inquiries can be directed to the corresponding authors.

## Ethics statement

The studies involving human participants were reviewed and approved by the Ministry of Science and Technology, Taiwan. Written informed consent to participate in this study was provided by the participants.

## Author contributions

J-CK and R-HK: conceptualization and validation and formal analysis. C-CC: methodology, software, investigation, and resources. All authors contributed to the article and approved the submitted version.
